# Surfactant-Free Synthesis and Scalable Purification of Triangular Gold Nanoprisms with Low Non-Specific Cellular Uptake

**DOI:** 10.3390/nano10030539

**Published:** 2020-03-17

**Authors:** Rafael Ramírez-Jiménez, Álvaro Artiga, Scott G. Mitchell, Rafael Martín-Rapún, Jesús M. de la Fuente

**Affiliations:** 1 Instituto de Ciencia de Materiales de Aragón (CSIC-Universidad de Zaragoza), c/ Pedro Cerbuna s/n, 50009 Zaragoza, Spain; raramjim@unizar.es (R.R.-J.); scott@unizar.es (S.G.M.); 2 Centro de Investigación Biomédica en Red in Bioingeniería, Biomateriales y Nanomedicina (CIBER-BBN), 28029 Madrid, Spain; 3 Instituto de Nanociencia de Aragón, Depto. Química Orgánica (Universidad de Zaragoza), c/ Mariano Esquillor s/n, 50018 Zaragoza, Spain

**Keywords:** gold nanoparticles, gold nanoprisms, gold nanoplates, localized surface plasmon resonance, non-specific cellular uptake, plasmonic nanoparticles, photothermal therapy

## Abstract

Gold nanoprisms possess remarkable optical properties that make them useful for medical biotechnology applications such as diagnosis and photothermal therapy. However, shape-selective synthesis of gold nanoprisms is not trivial and typically requires either toxic surfactants or time-consuming purification protocols, which can limit their applicability. Here, we show how triangular gold nanoprisms of different sizes can be purified by precipitation using the non-toxic glutathione ligand, thereby removing the need for toxic surfactants and bottleneck purification techniques. The protocol is amenable for direct scaling up as no instrumentation is required in the critical purification step. The new purification method provides a two-fold increased yield in gold nanoprisms compared to electrophoretic filtration, while providing nanoprisms of similar localized surface plasmon resonance wavelength. Crucially, the gold nanoprisms isolated using this methodology show fewer non-specific interactions with cells and lower cellular internalization, which paves the way for a higher selectivity in therapeutic applications.

## 1. Introduction

Anisotropic gold nanoparticles, such as nanorods (NRs) and nanoprisms (NPrs), possess remarkable optical properties [1]. By altering the size and the aspect ratio of NRs and NPrs, it is possible to tune their localized surface plasmon resonance (LSPR) across the near-infrared (NIR) region of the spectrum, the range of wavelength at which the absorbance of biological tissues is highly decreased [2]. This characteristic makes them promising for biomedical applications, in both therapy and diagnosis.

Besides the optical properties of nanoparticles, the way they interact with biomolecules and living matter is decisive for their performance in therapy. Although gold NRs are better photothermal transducers than gold NPrs, the latter are more effective at inducing cell death through photothermal ablation [3], due to their greater cellular internalization [3,4]. However, the superior cellular internalization of gold NPrs originates from non-specific interactions which can lead to poor cell specificity, resulting in unnecessary damage to local healthy tissue. It has been shown that usual stealth coating with poly(ethyleneglycol) (PEG) does not prevent the non-specific uptake of gold NPrs by cells [3,4,5] and no other better alternative has yet been reported for gold NPrs.

The synthesis of gold NPrs is more challenging than their isotropic counterparts and requires low metal concentrations with weak reductants. Various capping agents or templates such as polymers or surfactants are used to favor shape selectivity [6,7]. By far the most successful approach to gold NPrs [8,9,10,11] involves the use of cytotoxic surfactants cetyltrimethylammonium bromide (CTAB) or chloride (CTAC), but their toxicity is a serious limitation for applications involving direct contact of the material with healthy tissues [12]. CTAB-free preparation of gold NPrs typically involves mild reducing conditions in combination with a directing agent which can be other surfactants, synthetic [13,14,15,16,17,18] or natural [19] polymers, liquid crystals [20], or alkaline halides [21]. Spherical nanoparticle side-product should be removed since they consume precious active or vectorization molecules when bioconjugation steps are required and the presence of more particles could lead to a greater immune response and increase of toxicity. The options for isolating gold NPrs are time consuming and only affordable at very low scale, which hinders subsequent in vivo testing. Those methods include polyelectrolyte/micelle depletion flocculation [18], electrophoresis [21], dialysis [22], and shape selective deposition [23].

**Figure 1:**
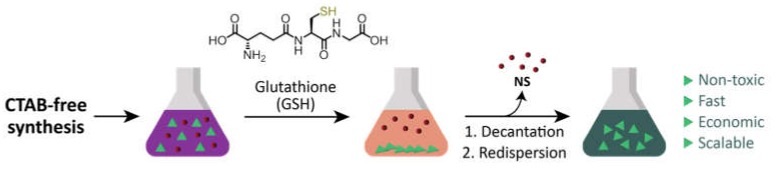
Proposed purification of gold nanoprisms (NPrs) from a mixture with gold nanospheres (NSs).

Here, we present a scalable separation method based on the selective quantitative precipitation of gold NPrs after a surfactant-free synthesis. Our hypothesis was that *zwitterionic* thiol-containing molecules, such as glutathione (GSH), could promote interparticle interactions [24,25] which would be more important in NPrs than in nanospheres (NSs) due to the larger flatter surface area of NPrs, which would favor multiple point interactions [6] (Figure 1).

## 2. Materials and Methods

### 2.1. Synthetic Method for the Nanoprisms with Plasmon Band at 1100 nm (1100NPr/NS) 

The protocol was adapted from Alfranca et al. [3]. Briefly, 200 mL of aqueous HAuCl_4_·H_2_O (Strem Chemicals, Newburyport, MA, USA) 2 mM (136 mg, 400 µmol) were mixed with 140 mL of Na_2_S_2_O_3_ (Sigma-Aldrich, San Luis, MO, USA) 0.5 mM (11 mg, 70 µmol) containing 10 μL of KI (Panreac, Barcelona, Spain) 0.1 M (0.16 mg, 1 µmol), dissolved in Milli-Q water from a Millipore Q-POD® system from EMD Millipore (Darmstadt, Germany). This thiosulfate addition was performed by pouring the thiosulfate solution into the gold solution in a slow but continuous way. After 4 min, another 140 mL of Na_2_S_2_O_3_ 0.5 mM (11 mg, 70 µmol) containing 10 μL of KI 0.1 M (0.16 mg, 1 µmol) were added. After another 4 min, 60 mL of Na_2_S_2_O_3_ 0.5 mM (4.7 mg, 30 µmol) were slowly added to the solution and the resulting mixture was left reacting for an hour.

### 2.2. Purification of the Nanoprisms with Plasmon Band at 1100 nm by Selective Precipitation with GSH (1100NPr-GSH)

To purify the nanoprisms from 1100NPr/NS by selective precipitation, a borate buffer 100 mM pH 8 was added (final concentration 10 mM) to the crude mixture of nanoprisms and nanospheres (1100NPr/NS) after the synthesis. Then, a solution of glutathione (GSH) in borate buffer 10 mM pH 8 with a ratio GSH:Au of 5:1 (in mg) was added to the nanoparticle dispersion. After that, the pH was raised to 12 with the addition of aqueous NaOH (2 M). Finally, the solution was left overnight without stirring. The next day, the supernatant, mostly containing NSs, was removed. The purified NPrs remained in the green precipitate and could be easily redispersed in water (1100NPr-GSH).

### 2.3. Preparation of 1100NPr-GSH-PEG

A solution of HS-PEG-COOH (aq.) was added to 1100NPr-GSH with a ratio PEG:nanoprisms of 2:1 (in mg). After that, the pH was raised to 12 with the addition of aqueous NaOH (2 M). Finally, the solution was sonicated for 1 h at 60 °C to complete the coating with PEG. The resultant nanoprisms were centrifuged at 5500 *rcf* for 15 min at room temperature to remove unreacted reagents and unwanted by-products. While the supernatant was discarded, the precipitate was resuspended in the same volume of water and two further washing steps were performed with Milli-Q water using the same conditions.

### 2.4. PEGylation Method for 1100NPr/NS and 900NPr/NS

To stabilize the nanoprisms, heterobifunctional HS-PEG-COOH (5 kDa) was conjugated to the gold surface. For this purpose, a solution of HS-PEG-COOH (aq.) with a ratio PEG:Au of 2:1 (in mg) was added to the nanoprisms. After that, the pH was raised to 12 with the addition of aqueous NaOH (2 M). Finally, the solution was sonicated for 1 h at 60 °C to complete the coating with PEG. The resultant nanoprisms were centrifuged at 5500 *rcf* for 15 min at room temperature to remove unreacted reagents and unwanted by-products. While the supernatant was discarded, the precipitate was resuspended in the same volume of water and two further washing steps were performed with Milli-Q water using the same conditions.

### 2.5. Purification of the NPrs-PEG with Gel Electrophoresis (1100NPr-PEG and 900NPr-PEG)

The aqueous dispersion of PEG-grafted nanoprisms and PEG-grafted nanospheres (2 mL, 1 mg Au/mL) was loaded (mixed with loading buffer, i.e., TBE 0.5x, 5% glycerol) in wells within an agarose gel (2.5%) immersed in an electrophoresis cuvette filled with TBE 0.5x. Electrophoresis separation was run at 120 V for 15 min (1100NPr/NS-PEG) and 10 min (900NPr/NS-PEG). Due to the higher electrophoretic mobility and lower hydrodynamic diameter of NSs compared to NPrs, the nanospheres entered in the gel and the nanoprisms stayed in the wells (Figure S2). At the end of the experiment, the nanoprisms were recovered from the wells with a micropipette.

### 2.6. Inductively Coupled Plasma Atomic Emission Spectroscopy (ICP-AES) for Gold NPs Concentration and Synthesis Yield Analysis

Samples were transferred to Eppendorf vials for acid digestion. To digest the samples, they were treated with 100 µL of *piranha* solution (3:1 vol/vol; sulfuric acid, 96%: hydrogen peroxide, 33%) for 15 min at room temperature followed by 300 µL of aqua regia (1:3 vol/vol; nitric acid, 65%: hydrochloric acid, 37%) for 2 h at room temperature. Subsequently, the samples were incubated at 60 °C for 15 min and diluted with Milli-Q water to 20 mL. All samples were prepared in triplicate and evaluated by ICP-AES (Horiba Yobin Activa atomic emission spectrometer with inductively –coupled plasma (Horiba Scientific, France) at the Central Analysis Service Bizkaia (Leioa, Spain).

### 2.7. Derivatization of NPrs with Gly-Arg-Gly-Asp-Ser (RGD peptide) (1100NPr-GSH-PEG-RGD)

1100NPr-GSH-PEG were derivatized with Gly-Arg-Gly-Asp-Ser (RGD peptide) (Sigma-Aldrich, San Luis, MO, USA) for promoting cellular uptake. Briefly, 0.5 mg of 1100NPr-GSH-PEG were incubated with 40 µg of EDC and 61 µg of Sulfo-NHS in 1 mL of MES buffer pH 6 for 30 min at 37 °C; activated 1100NPr-GSH-PEG were then incubated for 2 h at room temperature with 20 μg of RGD (42 µmol). Finally, 14 µg de tris(hydroxymethyl)aminomethane (120 µmol) were added to derivatize the remaining activated carboxylic groups for 2 h at room temperature; functional NPrs were then washed out of ligand excess by centrifugal precipitation; functional NPrs were centrifuged three times for 10 min at 6000 rpm, and then pellets were resuspended in Milli-Q water.

### 2.8. Optical Microscopy Internalization Studies

All NPrs suspensions were sterilized by filtering through 0.22 μm filters (CHMLAB, Barcelona, Spain) prior to addition to cell cultures. Vero cells were cultured at 37 °C in a 5% CO_2_ atmosphere in Dulbecco’s Modified Eagle Medium (DMEM) supplemented with 10% fetal bovine serum, 2 mM glutaMAX^TM^ and 100 U/mL of penicillin/streptomycin. For the preparation of the samples of fixed cells for dark-field microscopy visualization, 5 × 10^4^ Vero cells per well were seeded on a glass coverslide placed in a 24-well plate and grown overnight under standard cell culture conditions (37 °C; 5% CO_2_). The following day, NPs in DMEM at 50 µg/mL were added to each well and incubated for 24 h (V_f_ H_2_O < 10%). Cells were washed four-times with Dulbecco’s phosphate-buffered saline (DPBS), fixed in 4% paraformaldehyde for 20 min, washed twice with DPBS, and incubated for 10 min with of 4′,6-diamidino-2-phenylindole (DAPI) (3 µM) for nuclei labeling. The coverslips were mounted on glass microscope slides using 6 μL of Prolong^®^ Diamond Antifade Mountant from Life Technologies^®^ (Carlsbad, CA, USA).

### 2.9. ICP Internalization Studies 

All NPrs suspensions were sterilized by filtering through 0.22 μm filters (CHMLAB, Barcelona, Spain) prior to addition to cell cultures. The experiment was performed in triplicate for each material: 1100NPr-PEG, 1100NPr-GSH-PEG, 1100NPr-GSH-PEG-RGD, and the control without NPrs. For these assays, 5 × 10^4^ Vero cells/well were grown overnight under culture conditions (37 °C, 5% CO_2_) in 24-well plates. The following day, the culture medium was retired and 400 μL/well of nanoparticles in DMEM at 50 µg/mL of NPrs (just DMEM in the control experiments) were added and they were incubated 24 h under culture conditions. After that, the culture medium was collected with the non-internalized nanoparticles and saved measuring their volume. Cells were then washed four times, twice with 400 μL of DPBS and twice with 400 μL phosphate buffered saline (PBS). Thereafter, cells were incubated 5 min with 100 μL of trypsin at 37 °C and resuspended in 200 μL DMEM to inhibit the enzyme. The number of cells in each sample was quantified by using a Neubauer chamber.

Piranha solution (100 μL) was added to cell samples for digestion during for 15 min at room temperature, followed by the addition of 300 µL of aqua regia and 2 h digestion at room temperature. Subsequently, the samples were incubated at 60 °C for 15 min and diluted with Milli-Q water to 20 mL. The amount of gold was measured by ICP-MS. The total amount of gold (NPrs) in 20 mL corresponded to the total amount in a well and was divided by the number of cells counted in the well.

## 3. Results and Discussion

**Figure 2:**
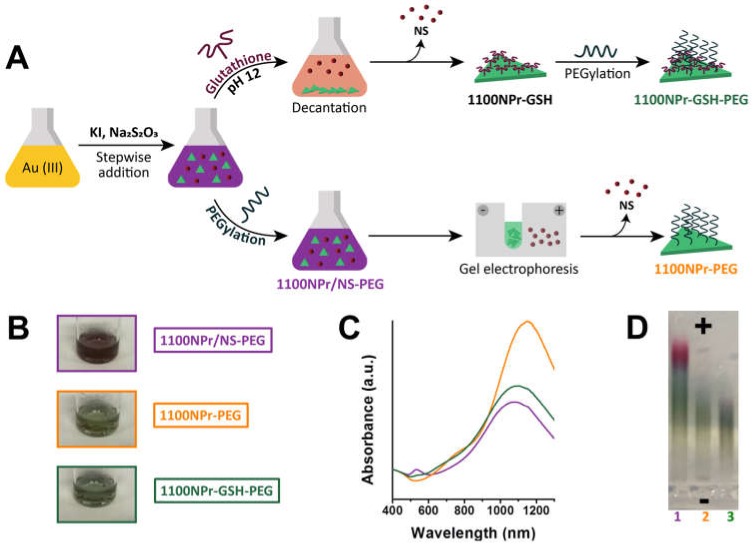
(**A**) Synthesis and purification of gold NPrs using two approaches: GSH-mediated deposition and electrophoresis separation. (**B**) Appearance of the NPr preparations. (**C**) UV-Vis-NIR absorption spectra and images of the NPr preparations for NPrs with LSPR band at ca. 1100 nm. (**D**) Electrophoresis gel comparison of the NP content between the three samples. Color code: 1100NPr/NS-PEG (▬), 1100NPr-GSH-PEG (▬), 1100NPr-PEG (▬).

Triangular nanoprisms with localized surface plasmon resonance wavelength (λ_LSPR_) at ca. 1100 nm were synthesized using a protocol that does not require any toxic reagent and can be adapted to tune the size and LSPR band of the NPrs [3,21,26]. Briefly, Au(III) solution was reduced with Na_2_S_2_O_3_ and KI forming a mixture (1100NPr/NS) of NPrs with LSPR at 1100 nm (1100NPr) and NSs (Figure 2). It has been reported that the as-synthesized mixture can be treated with heterobifunctional thiol containing PEG to stabilize the nanoparticles and to provide them with stealth properties. Conversely, we treated the as-synthesized mixture of NPrs and NSs (1100NPr/NS) with GSH at various pH values to promote interparticle interactions. At pH 3, addition of GSH led to the fast and irreversible aggregation of the particle mixture. When the GSH addition was performed at a higher pH, above the pK_a_ of both carboxylic groups and the isoelectric point of glutathione, NPrs deposition was mild and the mixture gradually separated into green sediment and purple-red supernatant. This fact was an indication that selective deposition of NPrs (green) was taking place. At pH 12, deposition occurred to a higher degree compared to pH 6 and 8 for the same amount of GSH and was the selected pH for the purification method. The sediment, formed by gold NPrs functionalized with GSH (1100NPr-GSH) was readily redispersed in water but a PEGylation step further enhanced the stability of the NPrs (1100NPr-GSH-PEG) and facilitated eventual bioconjugation (Figure 2). Importantly some GSH might remain on the surface filling the gaps left by PEG under certain conformations, and therefore providing a more efficient coating. When using the optimized conditions at pH 12, the UV-Vis-NIR spectrum of 1100NPr-GSH-PEG showed a complete suppression of the LSPR band of the NSs at 528 nm. On the other part, the LSPR band in the NIR region was completely suppressed in the supernatant indicating there was not a significant loss of NPr yield during purification (Figure S1).

In the literature, yields of NPs syntheses are often derived from absorbance spectroscopy measurements. In contrast, we determined the yield by more accurate ICP-AES on several 80-mg-scale syntheses of NPrs using the CTAB-free protocol and our GSH mediated deposition.

The obtained yield (Table 1, Entry 1) was comparable to the total yield (NPrs + NSs) reported for other synthetic methods and at least at a seven-fold quantity scale [22]. The NPrs could be recovered nearly quantitatively after the PEGylation step leading to 38 ± 7% (Table 1, Entry 2). For comparison, we also determined the yields after PEGylation of the as-synthesized mixture (1100NPr/NS-PEG) and the yield for an alternative, time-consuming, purification by gel electrophoresis (1100NPr-PEG) (Figure 2 and Figure S2). At 80-mg scale, the gel electrophoresis method led to a much lower 19 ± 3% yield of NPrs (Entry 4), which is just 50% of that obtained with the new method and using a labor-intensive protocol (typically 6 h).

The GSH-mediated deposition could also be used for the preparation of NPrs of other sizes. We demonstrated this by synthesizing pure 120 nm edge length NPrs with LSPR band at ca. 900 nm (900NPr). In this case, deposition was less effective and the amount of added GSH had to be increased. By doing so, a 24% gold yield precipitation was achieved (Table S1). The UV-Vis absorption spectrum of the supernatant displayed an LSPR band at 528 nm corresponding to the NSs, as well as a weak maximum at 815 nm attributed to the 900NPrs, indicating that some NPrs failed to precipitate (Figure S1). Nevertheless, the 24% overall yield represented a notable improvement on the 13% obtained using gel electrophoresis purification (Table S1). 

Taken together, both GSH-mediated deposition and electrophoretic filtration led to the apparent complete separation of the NSs by UV-vis-NIR (Figure 2); however, a significantly higher yield, two-fold increase, was obtained when the former was used. Additionally, GSH-mediated deposition is more amenable to scale-up than electrophoretic filtration, principally because it removes the need for costly instrumentation and workforce [27].

A priori, both methods could cause the enrichment in larger NPrs which would have an effect in the UV-vis-NIR spectrum. However, selective deposition did not lead to a shift in the LSPR (Figure 2), whereas the same band appeared red-shifted for 1100NPr-PEG indicating the lower yield with that method could be caused by size exclusion of smaller NPrs. Similar differences were observed for the NPrs with LSPR at 900 nm (Figure S1).

**Figure 3:**
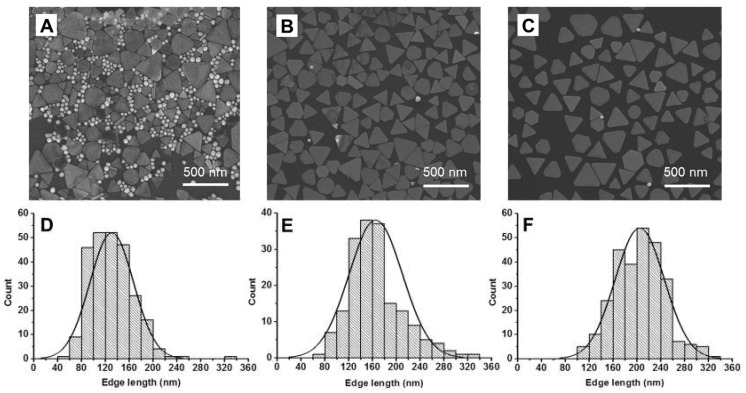
(**A**–**C**) Scanning electron microscopy (SEM) micrographs corresponding to 1100NPr/NS-PEG, 1100NPr-GSH-PEG, and 1100NPr-PEG, respectively; and (**D**–**F**) histograms corresponding to the triangular nanoprisms in 1100NPr/NS, 1100NPr-GSH-PEG, and 1100NPr-PEG preparations, respectively. Nanospheres were not taken into account for the histograms.

Scanning electronic microscopy (SEM) was used to verify that this methodology did not alter the particle size distribution and morphology of the NPrs (Figure 3 and Figure S3). The 1100NPr/NS-PEG samples contained a large amount of NSs, irrespective of the low intensity of the corresponding LSPR (Figure 3A). On the other hand, the selectivity of the GSH-mediated deposition was demonstrated by SEM images in which hardly any NSs could be detected (Figure 2B).

There was only a moderate increase in the dimension of the NPrs, which were 165 ± 45 nm edge length for 1100NPr-GSH-PEG (Figure 3B,E). On the other hand, 1100NPr-PEG samples showed significantly larger dimensions of 205 ± 43 nm (Figure 3C,F), as anticipated by the UV-Vis-NIR spectra (Figure 2C). The lower yield of the gel electrophoresis arose from needing a larger cut-off for the NPrs to eliminate all NSs. Comparatively selective deposition would be more sensitive to the shape of the NPs under our optimized conditions. Neither method could separate other anisotropic shapes produced during this synthesis, where approximately 10% corresponded to hexagons and other nanoplate shapes.

All particles gave stable colloidal suspensions in water, despite their low ζ-potential values. 1100NPr-PEG and 1100NPr-GSH-PEG exhibited similar values, −13.9 ± 0.5 mV and −15.9 ± 0.8 mV, respectively, pointing to an efficient coverage with PEG despite the presence of GSH. On the other hand, 1100NPr-GSH exhibited a higher value −23.0 ± 1.0 mV. 1100NPr-GSH-PEG stability compared favorably with our standard 1100NPr-PEG (Figures S4–S6). Figure S4 shows the time-dependent progression of the absorbance of the samples dispersed in phosphate buffers (20 mM) in the pH range 5–12 and in the presence of NaCl. 1100NPr-PEG were completely stable in water at room temperature but aggregated at pH below the pK_a_ of the carboxylate groups. Temperature and basic phosphate buffers caused the gradual aggregation of the NPrs over a few days (Figure S4B). Interestingly, 1100NPr-GSH-PEG presented higher stability. After 12 days, only half of the absorbance had been lost in NaCl and at pH 9 and 12 (Figure S4A).

An important step for assessing these particles for biotechnological applications involves evaluating their cytotoxicity and any cellular uptake. In principle, the size, shape, and PEG coating should determine the interaction of the gold nanoprisms for both 1100NPr-GSH-PEG and 1100NPr-PEG. However, we hypothesized that the presence of GSH in 1100NPr-GSH-PEG may modify the density of the PEG coating and the conformation of PEG molecules with respect to 1100NPr-PEG. Based on previous studies [3], a Vero kidney epithelial cell line was incubated with up to 100 µg/mL of AuNPrs. MTT cell viability assays showed that neither 1100NPr-PEG nor 1100NPr-GSH-PEG were toxic to the cells at any of the tested concentrations (Figure S7).

**Figure 4:**
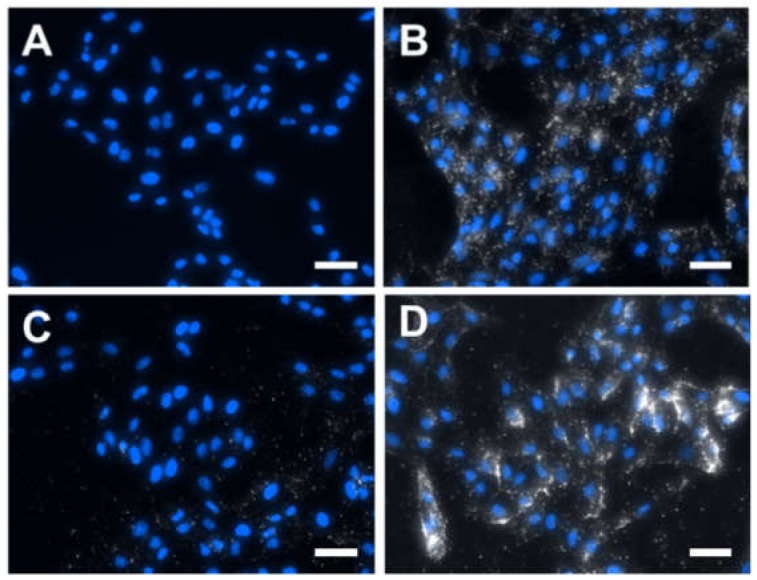
Dark-field microscopy images of the cellular internalization of AuNPrs. Cell nuclei were stained with 4′,6-diamidino-2-phenylindole (DAPI). (**A**) Vero cells as negative control, and treated with (**B**) 1100NPr-PEG, (**C**) 1100NPr-GSH-PEG, and (**D**) 1100NPr-GSH-PEG functionalized with a Gly-Arg-Gly-Asp-Ser (RGD) sequence (1100NPr-GSH-PEG-RGD). Concentration 50 μg/mL was used for all the NPrs. Scale bars correspond to 50 μm.

**Figure 5:**
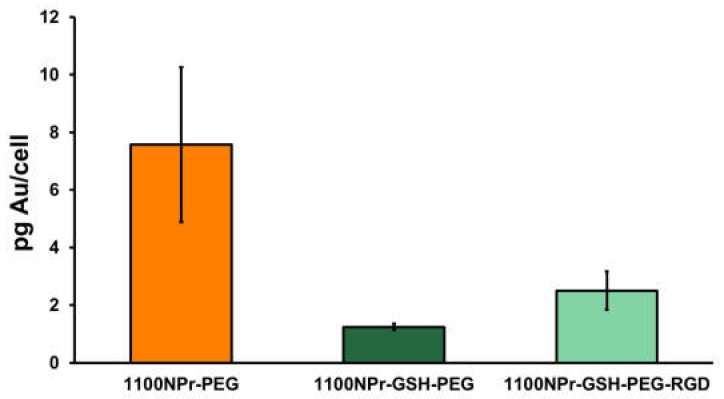
Amount of NPrs internalized by Vero cells after 24 h incubation with 50 µg/mL NPrs. Gold quantification was measured by inductively coupled plasma mass spectrometry (ICP-MS).

To study the cellular internalization of gold NPrs, Vero cells were incubated with 50 µg/mL of either 1100NPr-PEG or 1100NPr-GSH-PEG for 24 h and subsequently washed thoroughly to remove all the NPrs that were not attached/internalized by the cells. The cellular internalization of gold NPrs was then evaluated by dark-field microscopy/fluorescence (Figure 4) and by ICP analysis of the gold contained in the cell culture (Figure 5). By dark-field microscopy/fluorescence, in which cell nuclei were stained with DAPI, the presence of gold NPrs produces bright spots due to the more intense scattering of light (Figure 4). As previously reported, 1100NPr-PEG displayed significant cellular internalization (Figure 4B) [3,4,5]. In contrast, hardly any cellular uptake of 1100NPr-GSH-PEG could be observed (Figure 4C). From these images, a lower level of internalization of 1100NPr-GSH-PEG compared with the 1100NPr-PEG was evident, but not quantifiable. Quantitative ICP analysis confirmed this difference. The results show that while the concentration of 1100NPr-PEG internalized in Vero cells was equal to 7.58 pg Au/cell, the concentration of internalized 1100NPr-GSH-PEG was just 1.25 pg Au/cell, that is, about six times lower than that for 1100NPr-PEG (Figure 5).

This large difference in the uptake by cells could be explained due to the different coating that these nanoparticles have. The PEGylation of gold NPrs needs to be performed at a relatively low ionic strength with 5 kDa PEG to ensure the stability of the NPrs in the process. Under these conditions, dense packing (brush regime) is precluded by a hydrated coil conformation of the PEG molecules [28]. The presence of the glutathione ligand could allow a more effective coating of the gold surface, filling the gaps between the attachment points of the large PEG molecules. Further, glutathione negative charge could influence PEG conformation due to anionic repulsion. As a result, non-specific interactions are less important for 1100NPr-GSH-PEG than for 1100NPr-PEG leading to a decreased cellular uptake, which in turn could allow a higher selectivity in biomedical applications by including additional appropriate surface-functional ligands as targeting agents. To test this, we functionalized 1100NPr-GSH-PEG with a peptide with the motif arginine-glycine-aspartate (RGD) that promotes adhesion to cells via integrin-binding [29]. Furthermore, dark-field microscopy images revealed that functionalization with RGD led to a larger cellular internalization compared to 1100NPr-GSH-PEG (Figure 4D). The concentration of internalized 1100NPr-GSH-PEG-RGD was 2.50 pg Au/cell, which represents a two-fold increase on 1100NPr-GSH-PEG. These results clearly show that, in contrast to previous PEGylated NPrs, 1100NPr-GSH-PEG can be targeted with an appropriate functionalization strategy, while avoiding undesired non-specific uptake by non-targeted cells.

## 4. Conclusions

We report a new glutathione-based methodology to obtain triangular gold NPrs without the need for time- and materials-consuming purification procedures. At the tested 80-mg scale, triangular NPrs could be obtained in a high yield (42 ± 7%), free of spherical gold nanoparticles and without any partition of the batch, neither during the synthesis nor in the purification step. The yield of NPrs obtained was comparable to the total yield (NPrs + NSs) reported for other synthetic methods and at least at a seven-fold quantity scale [22].

The synthesis protocol allows the tuning of the LSPR band of the gold NPrs in the NIR biological window, at least from 900 to 1200 nm, making it relevant for a variety of biotechnological applications. We compared our method directly with previously reported gel electrophoresis separation and showed how purification with GSH was easier to scale up, and gave better yield and higher stability. Additionally, the GSH-separated nanoprisms show fewer non-specific interactions with cells that can be overcome with targeting or adhesive ligands paving the way for a higher selectivity in therapeutic applications. The higher cell selectivity and the scaled-up synthesis will greatly facilitate the use of nanoprisms in in vivo experiments. In the near future, we aim at studying the origin of the stealth properties of this material and to use it for specific targeted uptake by relevant cell lines.

## Figures and Tables

**Table 1:** Comparison of λ_LSPR_, yield, and size of the NPrs prepared by the two methods. ^1^

Entry	Material	λ_LSPR_ (nm)	Yield ^2^ (%)	Edge Length (nm)
1	1100NPr-GSH	1078	42 ± 7	188 ± 57
2	1100NPr-GSH-PEG	1092	38 ± 7	165 ± 45
3	1100NPr/NS-PEG	1076	66 ± 4	131 ± 37
4	1100NPr-PEG	1149	19 ± 3	205 ± 41

^1^ ICP-AES results; ^2^ percent gold yield based on the initial amount of gold(III) in the synthesis and extracted from the results of four syntheses.

## Supplementary Materials

The following are available online at https://www.mdpi.com/2079-4991/10/3/539/s1, Figure S1: UV-Vis-NIR spectra of the materials. Figure S2: Picture of an electrophoretic gel for the purification of gold nanoprisms. Figure S3: SEM results for 900NPr/NS-PEG, 900NPr-GSH-PEG and 900NPr-PEG. Figure S4: Comparison of the colloidal stability of 1100NPr-PEG and 1100NPr-GSH-PEG. Figure S: Colloidal stability test applied to 1100NPr-GSH-PEG. Figure S6: Colloidal stability test applied to 1100NPr-PEG dispersed. Figure S7: MTT cell viability assays results in Vero cells. Table S1: Yield comparison between the two methods by gel electrophoresis.
