# Supplementary material for: Surfactant-Free Synthesis and Scalable Purification of Triangular Gold Nanoprisms with Low Non-Specific Cellular Uptake
Source: Nanomaterials (Basel). 2020 Mar 17;10(3):539. doi: 10.3390/nano10030539 (PMC7153367; doi:10.3390/nano10030539)
Supplement: Supplementary file 1 [file nanomaterials-10-00539-s001.pdf]

Supplementary Material to

Surfactant-free synthesis and scalable purification of triangular gold nanoprisms with low cellular uptake

Rafael Ramírez-Jiménez [1,2], Álvaro Artiga [1,2], Scott G. Mitchell [1,2], Rafael Martín-Rapún [1,2,3,*] and Jesús M. de la Fuente [1,2,*]

1 Instituto de Ciencia de Materiales de Aragón (CSIC-Universidad de Zaragoza), c/ Pedro Cerbuna s/n, 50009 Zaragoza, Spain.
2 Centro de Investigación Biomédica en Red in Bioingeniería, Biomateriales y Nanomedicina (CIBER-BBN), 28029 Madrid, Spain.
3 Instituto de Nanociencia de Aragón, Depto. Química Orgánica (Universidad de Zaragoza), c/ Mariano Esquillor s/n, 50018 Zaragoza, Spain.
* Correspondence: rmartin@unizar.es (R. M.-R.), jmfuente@unizar.es (J.M.F.)

Received: date; Accepted: date; Published: date

Materials and Methods

Chemicals

Potassium iodide (KI), nitric acid 65%, hydrochloric acid 37%, sulfuric acid 96%, hydrogen peroxide 33% and tris(hydroxymethyl)aminomethane were purchased from Panreac®. HS-C_2H_4-CONH–PEG–O–C_3H_6–COOH (MW = 5000 g/mol, HS-PEG-COOH) was purchased from Rapp Polymere. 2-(*N*-morpholino)ethanesulfonic acid (MES) was purchased from Alpha Aesar. Hydrogen tetrachloroaurate (III) hydrate ($HAuCl_4{\cdot}H_2O$) was purchased from Strem Chemicals. Sodium hydroxide (NaOH), sodium thiosulfate ($Na_2S_2O_3$), glucose (4-aminophenyl β-D-glucopyranoside), glutathione (GSH), *N*-hydroxysulfosuccinimide sodium salt (sulfo-NHS), Gly-Arg-Gly-Asp-Ser (Ref: G4391-5MG) (RGD) and 1-ethyl-3-[3-dimethylaminopropyl] carbodiimide hydrochloride (EDC) were supplied by Sigma-Aldrich and used as received. Complete Dulbecco's modified Eagle's medium (DMEM™), phosphate-buffered saline (PBS) and Dulbecco's PBS (DPBS) supplemented with Ca^{2+} and Mg^{2+} were purchased from Lonza® (Basel, Switzerland). DMEM was supplemented with 10% fetal bovine serum, 5% glutaMAX™ and 5% penicillin/streptomycin prior usage on any cell culture. MTT (3-(4,5-dimethylthiazol-2-yl)-2,5-diphenyltetrazolium bromide) was purchased from Invitrogen™ (MA, USA). Prior to use in the synthesis of gold nanoprisms, all glassware was washed with *aqua regia* (1:3 vol/vol; nitric acid, 65%: hydrochloric acid, 37%) and rinsed thoroughly with Milli-Q water from a Millipore Q-POD® system.

Characterization techniques

UV-Vis-NIR spectra were acquired using a Cary 50 Probe® spectrophotometer from Varian or a Jasco V670. SEM images were collected using a field emission SEM Inspect F50 with an EDX system INCA PentaFETx3 (FEI Company, Eindhoven, The Netherlands) in an energy range between 0-30 keV. (FEI Europe, Eindhoven, Netherlands) working at 200 kV. To prepare the SEM samples NPrs solution (3 µL, 0.03 mg/mL) was cast onto a silicon wafer and allowed to dry. The edge length of the NPrs was measured using the ImageJ software and the corresponding histograms were obtained using OriginPro 9.0 software. The histograms were fitted to a Normal Distribution Curve using OriginPro 9.0 (Figures 3 and S3), which provided the mean edge length and the standard deviation shown in Tables 1 and S1.

For the determination of synthesis and purification yields, the gold concentration was measured by ICP–Atomic Emission Spectroscopy (AES) using an Optima 8300 (Perkin Elmer®, MA, USA) instrument. For the quantification of gold in the internalization studies, the samples were analyzed by ICP-Mass spectrometry (ICP-MS) using Elan DRC-e (Perkin Elmer®, MA, USA).

The optical density (λ = 555 nm) for the cell viability measurements was recorded using a *EL 800TM* Absorbance Microplate Reader (*BioTek, VT USA*).

All bright-field, dark-field and standard fluorescence images of the cells were obtained with an Eclipse Ti with focal-plane shutter (FPS) system from Nikon (Tokyo, Japan) equipped with a phase-contrast system, a dark-field visualization system and a 387 ± 11/447 ± 60 nm (DAPI) cube filter connected to NIS-Elements Microscope Imaging software.

Synthetic Method for the Nanoprisms with Plasmon band at 900 nm (900NPr/NS)

The protocol has been adapted from Alfranca et al.[3] Briefly, 200 mL of aqueous $HAuCl_4$ 2 mM (136 mg, 400 µmol) were mixed with 176 mL of $Na_2S_2O_3$ 0.5 mM (14 mg, 88 µmol) containing 20 µL of KI 0.1 M (0.33 mg, 2 µmol), dissolved in Milli-Q water. This thiosulfate addition was performed by pouring the thiosulfate solution into the gold solution in a slow but continuous way. After 4 minutes, another 176 mL of $Na_2S_2O_3$ 0.5 mM (14 mg, 88 µmol), this time without KI, were slowly added and the resulting mixture was left reacting for one hour at room temperature.

Purification of the nanoprisms with Plasmon band at 900 nm by selective precipitation with GSH (900NPr-GSH)

To purify the nanoprisms from 900NPr/NS by selective precipitation, a borate buffer 100 mM pH 8 was added (final concentration 10 mM) to the crude mixture of nanoprisms and nanospheres (1100NPr/NS) after the synthesis. Then, a solution of GSH in borate buffer 10 mM pH 8 with a ratio GSH:Au of 7:1 (in mg) was added to the nanoparticle dispersion. After that, the pH was raised to 12 with the addition of aqueous NaOH (2 M). Finally, the solution was left overnight without stirring. The next day, the supernatant, mostly containing NSs, was removed. The purified NPrs remained in the green precipitate and could be easily redispersed in water (900NPr-GSH).

Cell Viability Assays

All NPrs suspensions were sterilized by filtering through 0.22 µm filters (CHMLAB, Barcelona, Spain) prior to addition to cell cultures. Vero cell lines (kidney epithelial cells from African green monkey) were acquired from the American Type Culture Collection (CCL-81). Vero cells were cultured at 37 °C in a 5% CO_2 atmosphere in DMEM supplemented with 10% fetal bovine serum, 2 mM glutaMAX™ and 100 U/mL of penicillin/streptomycin. Cells were seeded at a density of 5×10^3 cells per well in a 96-multiwell plate and incubated under cell culture standard conditions. After 24h, the medium was replaced for fresh DMEN containing gold nanoprisms (25, 50, 75 or 100 µg/mL) and incubated for another 24 h under the same conditions. In order to quantify the viability of the cells by MTT assay the medium was removed and the cells were incubated with 200 µL of DMEM containing 10 µL of 5-mg/mL (3-(4,5-dimethylthiazol-2-yl)- 2,5-diphenyltetrazolium bromide) (MTT) solution in the dark under culture conditions for 90 min. During this time, yellow MTT dye is converted into violet formazan crystals by the NAD(P)H-dependent succinate dehydrogenase, a mitochondrial enzyme in living cells. Finally, the plate was centrifuged at 1250 rpm for 25 min; the supernatant was removed and the formazan crystals were solubilized with 100 µL of DMSO. After a proper resuspension of the crystals, the optical density at 555 nm was recorded using a plate reader. The cell viability percentage of each sample was calculated using the following formula, with 100 µL of DMSO as blank:

$$\%\ Cell\ viability = \frac{OD\ treated\ sample - OD\ of\ blank}{OD\ of\ control\ cell\ without\ NP - OD\ of\ blank} \cdot 100$$

UV-Vis-NIR spectra of the materials

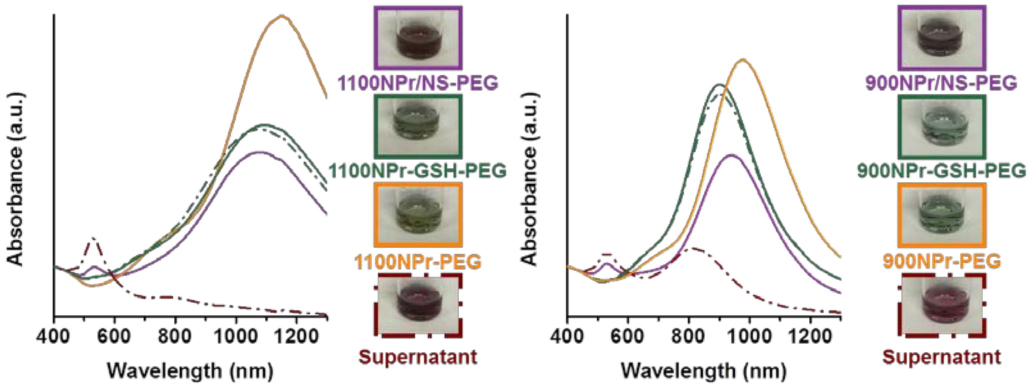

Figure S1. UV-vis-NIR absorption spectra and images of the NPrs preparation and purification. Left: 1100NPr/NS-PEG (—), 1100NPr-GSH-PEG (—), 1100NPr-PEG (—), sediment 1100NPr-GSH (—·) after treatment of the synthesis mixture with GSH, and the corresponding supernatant (—·). Right: 900NPr/NS-PEG (—),900NPr-GSH-PEG (—), 900NPr-PEG (—), sediment 900NPr-GSH (—·) after treatment of the synthesis mixture with GSH, and the corresponding supernatant (—·).

Purification of gold nanoprisms by gel electrophoresis

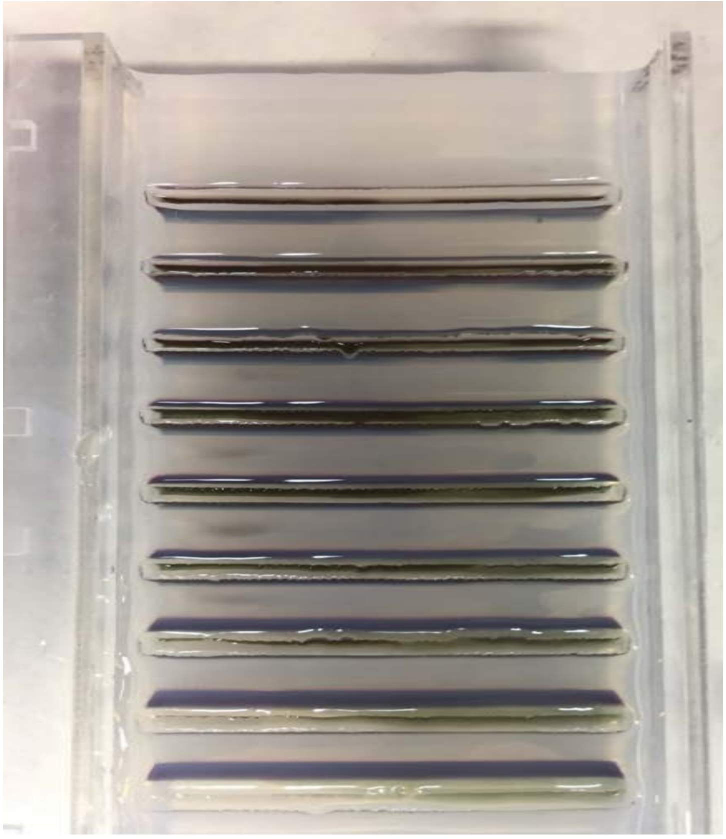

Figure S2. Purification of gold nanoprisms by gel electrophoresis: The aqueous dispersion of PEG-grafted nanoprisms and nanospheres was loaded in wells within an agarose gel (2.5%) immersed in an electrophoresis cuvette filled with TBE 0.5x. Electrophoresis separation was run at 120 V for 15 min (1100NPr/NS-PEG) and 10 min (900NPr/NS-PEG). Due to the higher electrophoretic mobility and lower hydrodynamic diameter of NSs compared to NPrs, the nanospheres entered in the gel and the nanoprisms stayed in the wells. At the end of the experiment the nanoprisms were recovered from the wells.

Yield comparison between the two methods

Table S1. Comparison of yield and λ_{LSPR} of the NPrs purified by the two methods.[1]

Entry	Material	λ_{LSPR} (nm)	Yield[2] (%)	Edge length (nm)
1	1100NPr-GSH	1078	42 ± 7	188 ± 57
2	1100NPr-GSH-PEG	1092	38 ± 7	165 ± 45
3	1100NPr/NS-PEG	1076	66 ± 4	131 ± 37
4	1100NPr-PEG	1149	19 ± 3	205 ± 41
5	900NPr-GSH	818	24	
6	900NPr-GSH-PEG	901	21	121± 19
7	900NPr/NS-PEG	898	48	110± 31
8	900NPr-PEG	940	13	125 ± 24

[1] ICP-AES results; [2] % gold yield based the initial amount of gold(III) in the synthesis and extracted from the results of four syntheses.

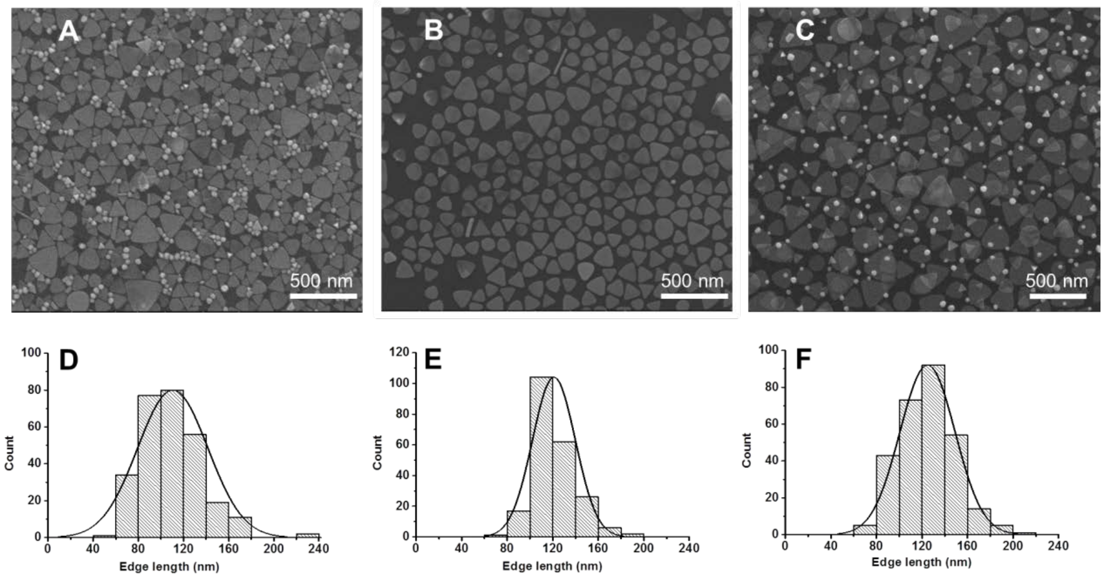

Figure S3. (A), (B) and (C) SEM micrographs respectively corresponding to 900NPr/NS, 900NPr-GSH-PEG and 900NPr-PEG. (D), (E) and (F) histograms respectively corresponding to 900NPr/NS, 900NPr-GSH-PEG and 900NPr-PEG preparations.

Stability of the nanoparticles

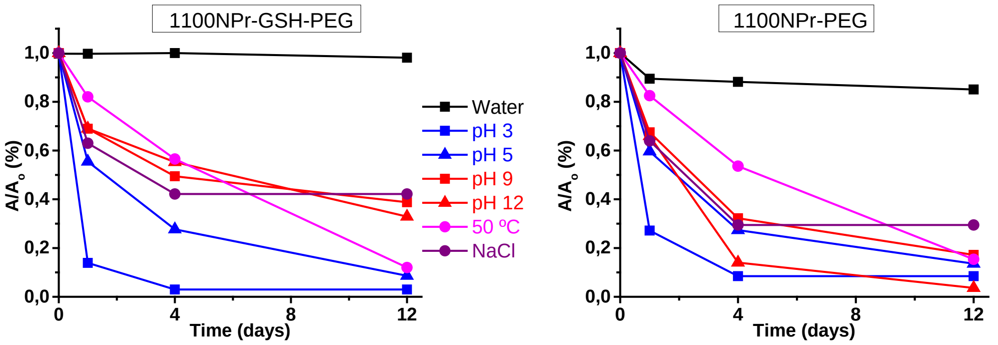

A B

Figure S4. Colloidal stability test applied to 1100NPr-GSH-PEG (A) and 1100NPr-PEG (B) dispersed in water at rt and 50 °C, in phosphate buffers (20 mM) at rt over the pH range from 3 to 13 and in the presence of 1 M NaCl. The absorbance relative to the initial value is represented versus time for all the samples.

Stability in buffers (1100NPr-GSH-PEG)

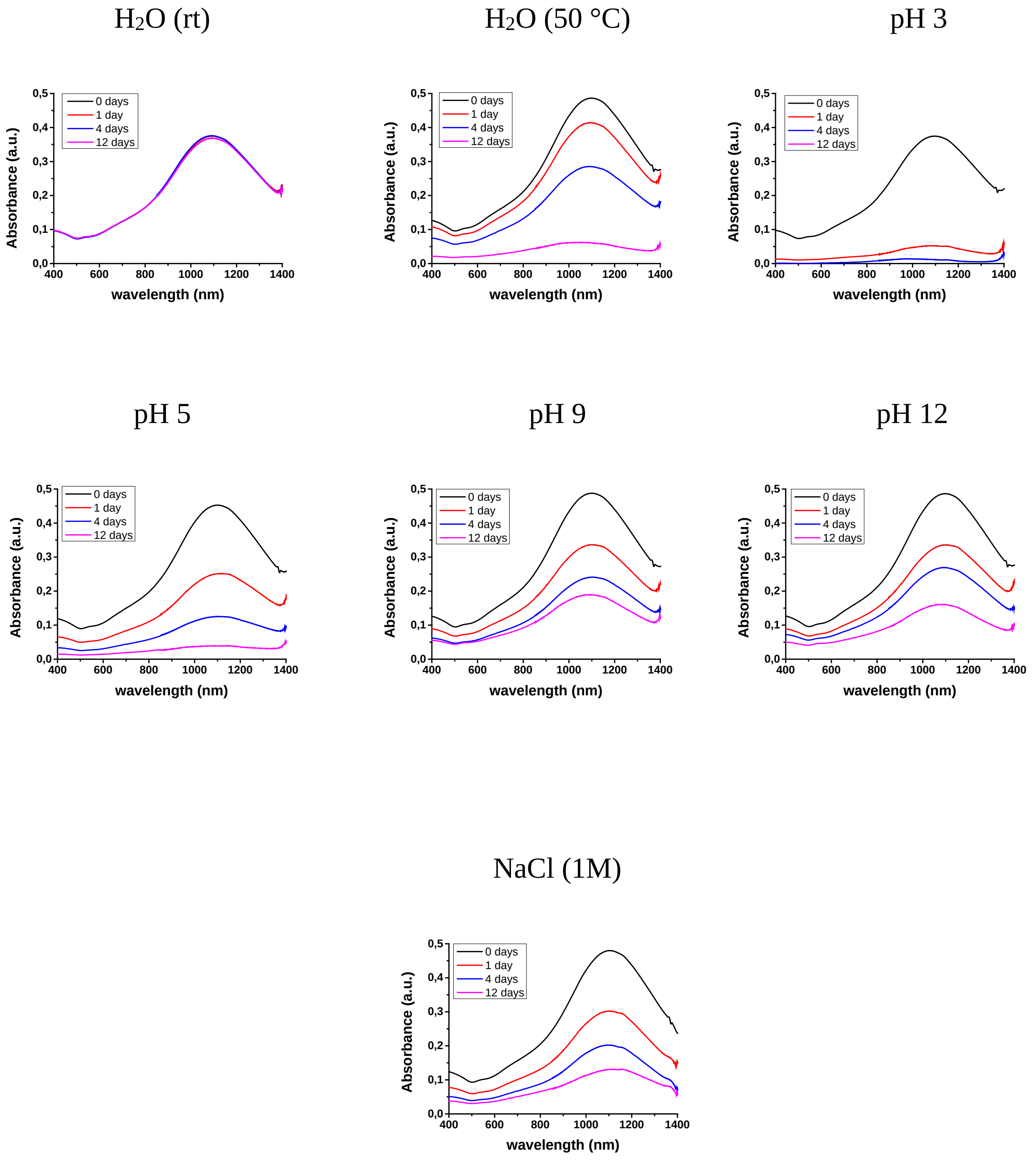

Figure S5. Colloidal stability test applied to 1100NPr-GSH-PEG dispersed in water at rt and 50 °C, in phosphate buffers (20 mM) at rt over the pH range from 3 to 13 and in the presence of 1 M NaCl.

Stability in buffers (1100NPr-PEG)

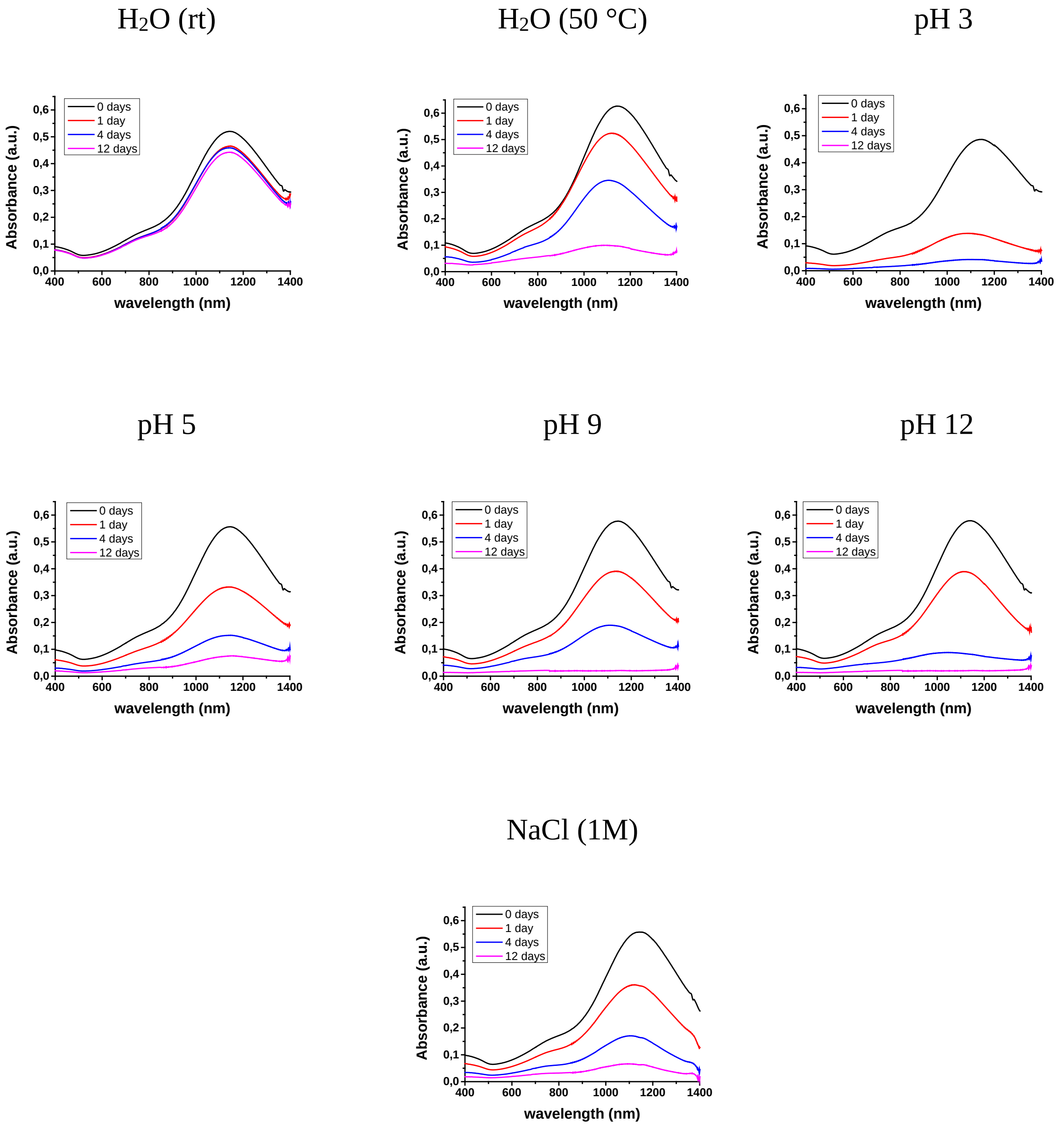

Figure S6. Colloidal stability test applied to 1100NPr-PEG dispersed in water at rt and 50 °C, in phosphate buffers (20 mM) at rt over the pH range from 3 to 13 and in the presence of 1 M NaCl.

MTT cell viability assays

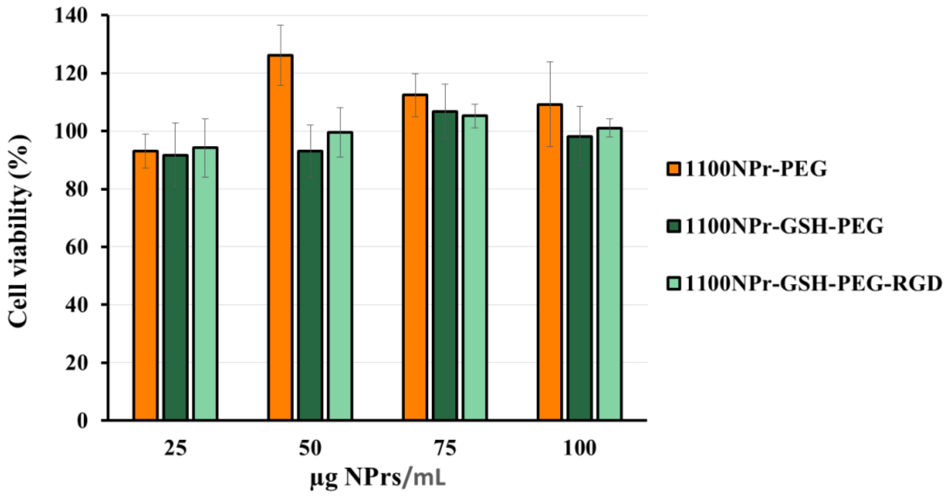

Figure S7. Cell viability of Vero cells after exposure with 100 µg/mL of NPrs for 24 h. Vero viability was the same compared with the cell control.
